# Percutaneous Patent Foramen Ovale Closure in Patients with Cryptogenic Stroke or Transient Ischemic Attack: A Retrospective Study

**DOI:** 10.1155/2022/2614225

**Published:** 2022-01-30

**Authors:** Yuan Liu, Yongming He, Pinjing Hui, Tan Li, Juehua Zhu, Caiming Zhao, Quanquan Zhang, Qi Fang

**Affiliations:** ^1^Department of Neurology, The First Affiliated Hospital of Soochow University, Suzhou 215006, China; ^2^Department of Neurology, Suzhou Ninth People's Hospital, 215200, China; ^3^Department of Cardiology, The First Affiliated Hospital of Soochow University, Suzhou 215006, China; ^4^Department of Stroke Center, The First Affiliated Hospital of Soochow University, Suzhou 215006, China

## Abstract

**Background:**

Patent foramen ovale (PFO) is associated with cryptogenic stroke (CS). Transcatheter closure of PFO is superior to pharmacotherapy for patients with CS or transient ischemic attack (TIA). More evidence is needed to evaluate the efficacy and safety of PFO closure in Chinese patients.

**Methods:**

This study enrolled ten CS patients and two TIA patients (mean age of 40.8 ± 9.7 y), including seven males (58%) and five females (42%) who underwent PFO closure in our center from January 2017 to July 2019. Baseline data, imaging data, and RoPE (Risk of Paradoxical Embolism) score were collected retrospectively. The preprocedural assessment and percutaneous transcatheter PFO closure were described in detail. The perioperative complications and follow-ups were recorded from all patients.

**Results:**

Among ten patients with CS, eight patients had a RoPE score of >6 and two patients had a RoPE score of 6. MRI confirmed multiple infarcts in seven cases, and infarct involving the cortex in nine cases. Abnormal ECG was found in three patients and abnormal Echo in four patients. Right-to-left shunt (RLS) was detected in all the patients by cTCD or cTTE. To be specific, RLS was observed in nine of the ten TEE-detected patients. No case had PFO complicated with atrial septal aneurysm (ASA). The success rate of PFO closure was 91.6%. No serious perioperative complications were observed. During a mean time of 26.5 ± 8 months (15-41 months) of follow-up, no recurrent cerebral infarction, TIA, or thromboembolism were detected in postoperative patients.

**Conclusions:**

PFO closure is safe and effective in the treatment of Chinese patients with CS or TIA.

## 1. Introduction

Cryptogenic stroke (CS) refers to the ischemic stroke that occurs in 30%-40% of patients with an unclear etiology [1–3]. Previous studies show that patent foramen ovale (PFO) is associated with CS, especially in patients aged less than 55 years, which approximately accounted for one half [2, 4–6]. Controversy exists over the preferred management strategy in preventing recurrent stroke for patients with CS and PFO. In 2017, the results of three multicenter randomized controlled trials (RCT) [6–8] showed that transcatheter closure of PFO is a better therapy in patients with CS than medical therapy (MT) alone. Multiple trial-level meta-analyses have confirmed the efficacy of closure for stroke prevention [9]. The current consensus is that the key to successful PFO closure is a comprehensive assessment to select the optimal candidates in patients with CS. High-risk of PFO ascribed to a substantial shunt size, atrial septal aneurysm (ASA), or hypermobility may be important determinants of the clinical benefit of the procedure [7, 8]. Patients with transient ischemic attack (TIA) should be assessed precisely to exclude a mimic before referring for PFO closure. Although a few observational studies have been reported [10, 11], more evidence is needed to evaluate the efficacy and safety of PFO closure in Chinese patients with CS or TIA.

## 2. Methods

### 2.1. Baseline Data Collection

In this study, data of 12 patients with CS or TIA, who underwent PFO closure in our center from January 2017 to July 2019, were retrospectively collected. Clinical baseline data included diagnosis, age, sex, history of hypertension, type 2 diabetes mellitus, and current history of smoking. Imaging data included contrast transcranial Doppler (cTCD), contrast transthoracic echocardiography (cTTE), transesophageal echocardiography (TEE), computed tomography (CT), and Magnetic Resonance Imaging (MRI). Risk of Paradoxical Embolism (RoPE) score was calculated for CS patients [12]. The diagnosis of ischemic stroke, TIA, and CS was based on the relevant literature and classification of the Trial of ORG10172 in Acute Stroke Treatment (TOAST) [13, 14]. This study was approved by the Independent Review Board (IRB) of Soochow University and was conducted in accordance with the principles of the Helsinki declaration.

### 2.2. Diagnosis of PFO and Preprocedural Assessment

The preprocedural assessment included, but not limited to the following: (1) Electrocardiogram (ECG) and Echocardiography (Echo) to exclude atrial fibrillation (AF) and evaluate the cardiac structure, (2) cTCD or cTTE or both to confirm the presence of right-to-left shunt (RLS) across the PFO at rest or application of Valsalva manoeuver, and (3) TEE to identify the size and anatomical characteristics of the PFO. In our center, RLS detected by cTTE and cTCD was graded according to the standards established in previous studies [15, 16].

### 2.3. Procedure of Percutaneous Transcatheter PFO Closure

Informed consent was obtained from all the patients. According to the results of TEE, all of the patients received the Amplatzer PFO occluder with a disc size of 28 mm. After local anaesthesia, a guidewire and a catheter were inserted through the femoral vein, the right atrium, and PFO, till to the left superior pulmonary vein. Then, the catheter was removed. Next, a transport sheath was advanced to the left superior pulmonary vein via the guidewire. Finally, the guidewire was removed and the transport was reserved. A 2-disc occluder (28 mm) was transported to the left superior pulmonary vein through the transport sheath. Under fluoroscopic and echocardiographic guidance, the left atrial disc was deployed into the life atrium. When the left disc neared the atrial septum, the connecting waist was released. With tension on the delivery cable, the sheath was pulled back and the right atrial disc was deployed. TEE was performed to confirm the position of the device and residual shunts. The pull-push test was done to ensure a right position of the device.

Finally, the closure device was released and the transport sheath was removed. Patients were transferred to their ward and monitored for the next 6 hours. The periprocedural complications were recorded. It took 40 to 60 minutes to complete the entire procedure.

### 2.4. Perioperative Management and Follow-Up

All patients were given aspirin 3-5 mg/(kg·d) and clopidogrel 75 mg/d orally, 48 hours before the closure of PFO. After the implantation of the closure device, a maintenance therapy according to Chinese guidelines [15] was administered (aspirin and clopidogrel for a minimum of 6 months). The events of recurrent ischemic stroke, TIA, severe bleeding, thromboembolism, and death as well as Modified Rankin Scale score (MRS score), which ranges from 0 to 5, were recorded for each patient by telephone or face-to-face interview during follow-up.

## 3. Results

### 3.1. Baseline Characteristics

The clinical baseline data of the 12 patients is shown in Table 1. All patients were less than 60 years old, with a mean age of 40.8 ± 9.7 y, including seven males (58%) and five females (42%). Two patients were diagnosed with TIA, and ten patients were diagnosed with CS. Six of the patients carried one high-risk factor for cerebrovascular diseases: three patients had a history of newly diagnosed hypertension, and three patients were smokers. No patients had diabetes. Ten CS patients had no previous history of cerebral infarction or TIA. Among them, eight patients had a RoPE score of >6 and two patients had a RoPE score of 6.

### 3.2. Evaluation of Acute Infarct on MRI

**Figure 1:**
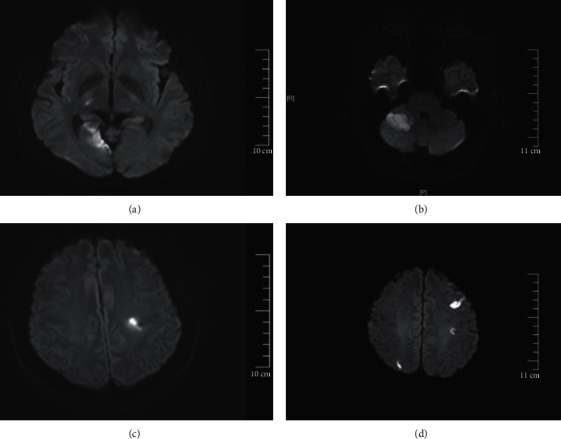
(a–d) Acute infarction in different brain regions of four patients on diffusion-weighted imaging (DWI) of MRI: right thalamus and right occipital lobe (a, patient ID: 1), right cerebellum (b, patient ID: 3), left frontoparietal junction, with a mild postinfarction hemorrhage (c, patient ID: 4), and left frontal lobe and right parietal lobe (d, patient ID: 7).

The diagnoses of acute ischemic stroke in ten patients were confirmed with diffusion-weighted imaging (DWI) of MRI. The location of acute infarct in each patient is shown in Table 2. Five cases had acute infarct in anterior circulation, and five cases had infarct in posterior circulation. Seven cases had multiple infarcts in different regions, and three cases had a single infarct in one region. The cortex was involved in nine patients. Mild postinfarction hemorrhage was found in one case (patient ID: 4, Table 2). The acute infarct on DWI of four of these patients is shown in Figure 1.

### 3.3. Evaluation of ECG and Echo

Out of twelve patients, ECG was found normal in nine patients, sinus bradycardia in two patients, and complete right bundle branch block in one patient. Echo was normal in eight patients and abnormal in four patients: one patient with mildly thick left ventricular wall, one patient with dilated aortic sinus, one patient with thick ventricular septum, and one patient with abnormal inferior and posterior wall movements.

### 3.4. RLS and Anatomical Structure of PFO

**Figure 2:**
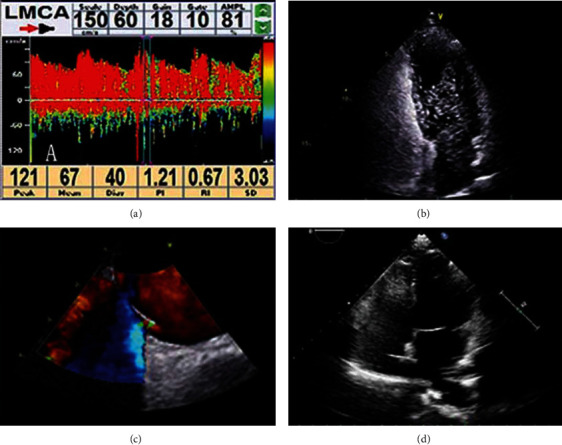
(a) Contrast transcranial Doppler (cTCD) detected large right-to-left shunt in a patient with CS; (b) contrast transthoracic echocardiography (cTTE) showed a large number of microbubbles in the left atrium within three cardiac cycles after the contrast entered the right atrium; (c) transesophageal echocardiography (TEE) detected right-to-left shunt and the width of gap (the yellow arrow) between the atrial septum and PFO valve; (d) contrast transthoracic echocardiography (cTTE) showed no microbubbles in the left atrium after PFO closure.

RLS was confirmed in all twelve patients by cTCD or cTTE or both before PFO closure. cTCD revealed large RLS in ten patients and moderate RLS in one. cTTE revealed large RLS in eight patients and moderate RLS in one (the same case to that detected by cTCD). TEE was completed in ten patients, all of which showed an imbricated structure and a residual flap channel between the primary and secondary septum in the middle part of the atrial septum. On TEE, RLS was detected in nine patients and absent in one patient. The PFO measured 0.8-2.1 mm in diameter, with three patients having a PFO diameter ≥ 2 mm. No case of PFO complicated with an ASA was found in this study. RLS detected in different examinations during preprocedural assessment of PFO closure (a–c) and no RLS detected by cTTE after PFO closure (d) are shown in Figure 2.

### 3.5. Success Rate and Complications of PFO Closure

PFO closure was completed successfully in eleven patients, with a success rate of 91.6%. The procedure could not be completed in one patient (patient ID: 8, Table 1) because we were unable to pass the guidewire through the foramen ovale. No serious complications were observed in the perioperative period, such as pericardial effusion, peripheral vascular injury, deep venous thrombosis of the lower extremities, perforation of the heart, valve regurgitation, displacement of the occluder, malignant arrhythmia, or death. TEE was performed immediately after the device was implanted, and no residual shunt was detected.

### 3.6. Results of Follow-Up Visit

The results of follow-up visit are shown in Table 3. All of the eleven patients who completed the PFO closure finished the follow-up visits, with a mean time of 26.5 ± 8 months (15-41 months). None showed recurrent cerebral infarction, TIA, thromboembolism, cerebral hemorrhage, gastrointestinal bleeding, chest tightness, or chest pain. Nine patients with CS recovered well with an MRS score of 0-1. In the follow-up period, seven patients underwent RLS reexamination by cTTE: two at six months, four at seven months, and one at fifteen months, respectively. Residual shunts were all not detected in the seven patients. Six patients continued the use of aspirin for secondary prevention of ischemic stroke while other five patients discontinued it.

## 4. Discussion

This retrospective study indicates that the closure of PFO is safe and easy to perform in patients with CS or TIA. No serious complications were observed during a mean follow-up of 26.5 months. The key point is that the overall evaluation should be performed in all of the patients, which will be helpful to identify CS or TIA related to PFO before the intervention.

The prevalence of PFO is 20-25% in the general population [17] and nearly 40% in patients with CS [18]. Three possible mechanisms were proposed in PFO-related ischemic stroke: paradoxical embolism, in situ thrombosis of PFO, and arrhythmia [19]. A series of observational studies show that the closure of PFO reduces the recurrence of ischemic stroke in patients with CS. However, CLOSURE I [1], PC [20], and RESPECT [21] trials failed to demonstrate the superiority of PFO closure over antithrombotic therapy. Until 2017, the results of CLOSE [7], Gore REDUCE [8], and Long-Term Outcomes of RESPECT study [6] showed that PFO closure significantly reduced the recurrence rate of ischemic stroke in patients with CS. Multiple trial-level meta-analyses have confirmed the efficacy of closure, yielding an odds ratio (OR) of 0.44 compared with MT alone [9, 22–24]. Subgroup analyses showed the benefit of PFO closure was significant in patients with PFO associated with substantial RLS or ASA [25]. Besides, it did not increase the incidence of adverse events, such as major bleeding or death.

PFO is associated with CS in patients younger than 55 years old (OR = 5.1) [4]. In these five randomized controlled trials [1, 7, 8, 20, 21], the mean age of the patients was 42.2-46 years. All of the 12 patients in our study were <60 years old, 91.6% of whom were <55 years, with a mean age of 40.8 years. The RoPE score was used to evaluate the association between PFO and CS [12], with a PFO-attributable fraction of 0% for 0–3 points, 62% for 6 points, and 88% for 9–10 points [12]. In this study, six patients had one cerebrovascular risk: hypertension in three and smoking in three. The RoPE score was >6 in eight patients and was 6 in two patients. Therefore, the inclusion criteria of PFO closure for patients with CS was stricter in our center, and these patients were more likely to benefit from the PFO closure.

Infarcts in the posterior circulation and multiple small infarcts in the cortex are found to be more common in patients with CS [26]. According to the MRI results in this study, 7 of the 10 patients with CS had multiple infarcts in different regions, and 9 of them had infarcts involving the cortex. This involvement has been taken as an index to determine stroke related to PFO [12]. Next, 50% of the patients developed infarcts in the anterior circulation and the remaining 50% in the posterior circulation, which showed no distributional difference, may be related to the small sample size of this study.

The REDUCE study [8] shows that among patients with PFO-associated CS, the benefit of PFO closure is most remarkable in those with large RLS or ASA. The CLOSE [7] study included only the patients with moderate to large RLS of PFO; recurrent ischemic stroke was not reported in any case of the closure group during an average follow-up time of 63.6 months. It was reasonable that endovascular closure can protect only against the PFO-associated strokes; thus, identifying the candidates to benefit from the endovascular procedure of the “mechanical vaccine” is very essential. Stringent selection may include CS patients rigorously ruling out alternative etiologies, younger patients without risk factors and with superficial infarcts, and patients with PFO and large RLS and/or ASA [25]. In the current study, all of the 12 patients screened by cTCD or cTTE showed moderate to large RLS, which was consistent with the CLOSE study. However, complex PFO with ASA was not found in this cohort, the proportion of which was high (32.8%) in the CLOSE study [7]. Also, 53% of patients included in the DEFENSE study in Korea had moderate to large RLS [27], which was consistent with the CLOSURE study [1]; however, the percentage of patients with an ASA was significantly lower in the DEFENSE study than in the CLOSURE study (10% vs. 36.6%). We suspected that could be probably due to the lower incidence of an ASA in PFO patients in Asia than in Europe and the United States, which need more epidemiological data to be investigated in the future. In our group, RLS was detected in all the patients by cTCD or cTTE; however, among ten patients confirmed RLS by TEE, RLS was not detected in one patient, which may be related to the lower sensitivity of RLS detected by TEE than that by cTTE [28].

The success rate of PFO closure in this study was 91.6%, with a fast recovery and short hospital stay. Amplatzer occluder device was implanted in 11 patients; we did not observe any perioperative complication. No residual RLS was found on reexamination with TEE performed immediately after the procedure. New-onset AF is the commonest complication after PFO closure, with an incidence of 2.9%-6.6% [29, 30]. AF mainly occurs within 30 days after the device implantation, possibly due to local inflammation after device implantation, irritation or stretching of the interatrial septum during device deployment, or the specific use of an umbrella-clamshell device [9]. As reported, 72% of new-onset AF recovered within 45 days spontaneously and would not increase the risk of ischemic stroke [1, 7, 31, 32]. In our study, one patient developed supraventricular tachycardia on the 9th day of implantation, but the sinus rhythm was restored by treatment with amiodarone, and dexamethasone was used for its anti-inflammatory action. No arrhythmia occurred during the 10 months of follow-up. A review of clinical data of this patient before PFO closure revealed the sinus rhythm with complete right bundle branch block on ECG, and motion abnormalities in the inferior and posterior wall on Echo, but no history of myocardial infarction and congestive heart disease was present. Thus, it may be indicated that patients with cardiac structural abnormalities or abnormal ECG changes may have a higher incidence of arrhythmias after PFO closure. Studies indicate potential risk factors for postclosure AF include older age, large devices with a disc size > 30 mm, large ASA, or low RoPE score < 69.

So far, no consensus has been reached about the duration of antiplatelet therapy after PFO closure. The majority of previous studies have insisted on using dual antiplatelet therapy for 1-6 months, followed by a monoantiplatelet therapy for six months-two years [1, 6, 7, 15, 20]. In our center, the antiplatelet therapy after PFO closure was provided as per the Chinese guidelines [15]. During our follow-up, all of the eleven patients received antiplatelet therapy. Among them, four patients maintained oral aspirin for secondary prevention of cerebral infarction after six months of PFO closure.

In summary, this retrospective observational study analyzed the process, efficacy, and safety of PFO closure in 12 patients with CS or TIA. It proved that PFO closure is safe and effective in these patients, which is coincident with previous randomized control studies. On the other side, the overall evaluation should be fully performed to identify PFO-related CS or TIA before the intervention. There are some limitations to our study. First, we did not compare the efficiency of PFO closure to that of antiplatelet therapy or anticoagulation therapy for secondary prevention of recurrent stroke or TIA. Second, the proportion of patients who were reexamined for RLS by cTTE or cTCD during the follow-up period was relatively low (7/11), so the efficacy of PFO closure and the recurrence of a RLS cannot be fully evaluated in a long-term follow-up. In the future, multicenter RCT could be designed to obtain more evidence about the efficacy of PFO closure for Chinese patients with CS or TIA.

## Figures and Tables

**Table 1:** Baseline characteristics of 12 patients with PFO and CS or TIA.

Patient ID	Sex	Age	HBP	DM	Smoking	Diagnosis	RoPE score
1	F	46	-	-	-	CS	8
2	F	56	+	-	-	CS	6
3	M	44	-	-	+	CS	6
4	F	37	+	-	-	CS	8
5	M	37	-	-	-	CS	8
6	M	26	-	-	-	CS	10
7	M	48	+	-	-	CS	7
8	F	36	-	-	-	CS	9
9	M	25	-	-	+	CS	9
10	M	37	-	-	+	CS	8
11	F	54	-	-	-	TIA	/
12	M	44	-	-	-	TIA	/

The RoPE score was calculated for 10 patients with CS. The total score was 10 points: 1 point for hypertension, diabetes, smoking, or previous TIA/stroke; 1 point for cortical infarction; 0-5 points for different age groups (5 points for 18~29 years, 4 points for 30~39 years, 3 points for 40~49 years, 2 points for 50~59 years, 1 point for 60~69 years, and 0 points for ≥70 years) [12]; CS = cryptogenic stroke; TIA = transient ischemic attack; F = female; M = male; HBP = hypertension; DM = diabetes mellitus; “+” = the positive history; “-” = the negative history; “/” = rope score was not calculated for TIA patients.

**Table 2:** Location of acute infarct in 10 CS patients with PFO.

Patient ID	Sex	Age	Location of acute infarct
1	F	46	Right thalamus and right occipital lobe (P)
2	F	56	Left parietal lobe and left corpus callosum (A)
3	M	44	Right cerebellum (P)
4	F	37	Left frontoparietal junction (A)
5	M	37	Brain stem (P)
6	M	26	Right basal ganglia, lateral ventricle, and right frontal lobe (A)
7	M	48	Left frontal lobe and right parietal lobe (A)
8	F	36	Left frontal, parietal, and temporal lobe (A)
9	M	25	Right cerebellum and bilateral occipital lobes (P)
10	M	37	Left hippocampus and left occipital lobe (P)

F = female; M = male; P = posterior circulation; A = anterior circulation.

**Table 3:** Results of follow-ups in eleven patients after PFO closure.

Patient ID	Follow-up (month)	Current antiplatelet therapy	mRS score	Residual RLS detected by cTTE after PFO closure
1	41	Aspirin	0	No RLS detected after 15 months
2	40	Aspirin	1	/
3	28	No	0	No RLS detected after 7 months
4	27	No	1	No RLS detected after 7 months
5	25	Aspirin	1	No RLS detected after 6 months
6	24	No	0	No RLS detected after 7 months
7	22	Aspirin	0	No RLS detected after 6 months
9	17	Aspirin	1	/
10	15	Aspirin	0	/
11	27	No	0	No RLS detected after 7 months
12	26	No	0	/

“/” represents: RLS reexamination by cTTE was not performed on these patients during follow-up. Patient ID 8 was absent because the PFO closure procedure could not be completed in this patient.

## Data Availability

The patients' information listed as figures or tables used to support the findings of this study is included within the article. If more detailed information is needed, please contact Doctor Quanquan Zhang. Her email address is zhangquanquan@suda.edu.cn.
